# Chit-Chat

**Published:** 1880-04

**Authors:** 


### Editorial Chit-Chat.

MR. W. H. NICHOLAS has quit the Lehigh Valley Railway, and has opened a hat store in Wilkesbarre. He means to run his store as successfully as he did his train, in which undertaking his many friends wish him abundant patronage.

IN THE WOODS.—The July number of the BISTOURY is usually a week or two late, by reason of our annual vacation in the woods, which occurs in June. Do not be impatient therefore, if the next number does not come promptly on the first of the month.

NOT HALF.—More than one-half of our subscribers have failed to send in their subscriptions up to this time. We write for fun, enjoy publishing so excellent a journal for such a trifling cost—and all that, but would you not better pay for it and so share just a trifle of the attending expense ?

ELRIDGE PARK.—Elmira has a reputation for enterprise second to no other city in the State. She needs a public park, and as one stands begging at our very doors to be adopted (at a cost easily within our means), it is not in keeping with our reputation not to assume its ownership. When the Park was open and its use freely extended to the public, thousands of people were attracted to it from neighboring towns. Excursions of people were found almost daily enjoying its cool shade and attractive surroundings. Then, people spent much money with our tradesmen and so directly benefitted the city. Every merchant will testify to the increased patronage that came to him from these visitors to the Park.

Mr. F. G. Hall, than whom a more level-headed business man cannot be mentioned, advocates the leasing of the Park for a term of years, at a cost of about $2,000 per year. He says the city can well afford to invest that sum in keeping up the Park for the benefit that will come to us through increased visitations of non-residents, as well as affording a place of recreation for our own citizens. Mr. Hall is foremost in considering any project likely to be of benefit to our city. He has been advocating the leasing of the Park among our business men, and presents arguments that should have convincing weight with the city rulers. By all means, let us have our Park again.

STOP IT—STOP IT.—Stop writing us about that microscope which we offered for sale in the last BISTOURY. It was sold long ago. Almost as soon as the advertisement was read. We have no more microscopes for sale—don't ex pect to have any, and blamed if we ever want to advertise one again, if we are to receive a hundred or more inquires about it.

THE CONTRIBUTIONS.—We call attention to the excellence of our contributions. Is this not a large dose for the small sum of fifty cents ? The medical items, in this single number, are taken and condensed from medical journals, the aggregate cost of which would be fifty dollars a year! We desire our professional friends to take this into consideration and aid us in extending our circulation among physicians.

THE DAILY ADVERTISER.—Since our last issue, our morning paper has adopted a new dress and form, which we cannot permit to go unnoticed, notwithstanding the time that has elapsed since the change. The *Advertiser* has always been an excellent journal, and its position among the very best dailies of the land so universally recognized, that praise of its merits seems to be superfluous. It has been changed from a four to an eight page journal, its edges trimmed, and the leaves held in position in a substantial manner, making a perusal of its contents convenient and at all times pleasant. We rejoice in the evidence of its increased prosperity as evinced in its enlargment and improvement in form. May it continue to be, as it always has been in the past, one of the foremost dailies of our State.

THAT HOME.—"Don't forget to mention our home," said a lady to us through the telephone, this morning. The home referred to is designated the "Home for the Aged," and has been built by a benevolent bevy of ladies of this city, in which to care for the enfeebled old of both sexes. The Home is situated upon the broad avenue leading from the city to Horseheads; is a substantial two story, brick edifice, just completed and ready to be furnished. It is intended to receive and care for any aged people, of slender means, who prefer the comforts and company to be found in such an institution to that which their own scanty means might afford. The ladies hope to have it furnished and ready for occupancy by the first of June. A splendid opportunity is here offered for our people to aid them in securing carpets, furniture, crockery and other household appliances. It has been suggested that every church in the city furnish and maintain a room in the institution. This certainly would be an effective and commendable way of dispensing charity.

THE TELEPHONE.—Our city has one hundred subscribers to the Telephone Exchange. We can order supplies from our grocer, butcher, baker, druggist and other tradesmen without the inconvenience of calling at their places of business. We can converse with our friends in different parts of the city, at any hour of the day, and summon the family physician when his services are needed. It is a wonderful convenience, that grows in usefulness as the number who avail themselves of it increase. It does not seem difficult to foresee that the time is rapidly approaching when the Elmira Exchange will be connected with that of New York, Philadelphia, and other cities, so that we will be in conversational communication with our friends there. It is not at all improbable that within five years, the New York merchant will be in communication with, and receive orders from his patrons from all over the country, by telephone. It seems plain that the patronage of the mail service will be greatly decreased, and that men will write fewer letters and talk more with their customers through the telephone. This is a wonderful age, its attainments startling and its capabilities unfathomable, its——there rings the telephone bell, pardon the abrupt termination of our reverie.

FRITZ GETS TIRED OF BEING GOOD.—In response to numerous inquires about Fritz, we can only inform his friends that he in no wise lacks in affording abundant incidents in his daily life that could not fail of interesting fathers and mothers who have to deal with the same sort of an imp. Recently, his parents made a visit to a neighboring city. They announced that they would be gone one week, and promised Fritz numerous presents if he would be "good" while they were gone, and cause no complaints to be entered against him from the housekeeper. Fritz promised, and entered into the contract with a determination to win. The housekeeper reported that he was a wonderfully good boy—exceeding her wildest anticipations. He never teased the baby once. Was careful not to let the saw-dust out of any of her dolls, and did not swap his marbles to her for sugar-plums. He was in promptly to his meals—never was late to school—had no complaints entered from the boy next him on the pin business—regarded his grandma's apple barrel as sacred—avoided the cookie-can as though it were a castor oil receptacle—was not saucy to the cook—reported himself before dark, and never failed to go to bed at half-past seven, without being told. In short, he was a perfect model of a boy, for a straight week. Upon the evening when the week expired, Fritz went to bed as usual, remarking as he went toward his room—"Well, time's up!" In the morning, he was seen rushing down stairs with baby's doll by one leg; he scrambled through the parlor, upset nurse's mending basket, and put a sad fracture in it with his copper-toed shoes; then darted through the kitchen and shied the doll at a brindle cat that lay half snoozing, on the fence top, in the morning sun.

"Consarn that old cat, she thought I darsen't stir her up agin, but I'll jess let her know that time's up, and things ar boomin!"

"Why Fritz what *are* you doing?" cried the nurse.

"Doin—doin nothin—that's what! Ye don't expect a feller to be good for *ever*, do ye? I'm *tired* of being good, that's what I am—and time's up, and I can't stand it no longer. Whoop!" and away he rushed with his grandfather's best cane, to join in a game of shinney with a lot of dirty urchins down the street.

---

THE MONUMENT.—Doubtless our friends at a distance are anxiously watching the progress of the Adam Monument project. Just now, the committee are busy trying to select a suitable design. As yet they have had but six offered—three plaster casts and three drawings. All of them are more or less artistic, but nothing has been yet offered that fully satisfies them. The difficulty seems to be in presenting a nearly nude figure that shall at once indicate whom it is intended to represent, without an accompanying card of explanation. Said a Philadelphia sculptor to us: "You may stand a nude figure on a pedestal and it may represent John Smith or any other fellow—it stands for no one in particular." And just there is the trouble. The designs so far received have the apron of fig leaves, the serpent, apple, and Eve herself variously employed to aid in the identity of the chap poised on a globe. One design represented a finely developed man, with neatly trimmed hair and whiskers—(where in fury did he get his scissors?) with a party-dress of fig leaves. He stood upon a winged world, (the wings somehow representing the protecting care of God,) a serpent entwining the sphere, (the venomous beast being emblematic of how sin has encircled the earth,) and in his hand he held a fine, luscious pomegranate, extending it toward you as though inviting you to take a bite, as much as to say, "Eve played that little game on me and now I'd like to try it on you." The man is perfect in form save that he has no umbilicus, a delicate reminder that, as he was never *born*, he could not have had any use for one.

The best figure that has so far been presented, came from a Washington sculptor, and represents a splendid specimen of the genus homo, gracefully poised upon a sphere, his weight resting upon his left foot while the right is slightly advanced. His arms lay across his chest, bringing his clasped hands in a line with the left shoulder. His face is turned toward the right with an expression of joyous surprise, as though he had just obtained a view of Eve for the first time. [The last named member of the committee thought this sentiment could have been better conveyed by having Adam represented in trying to jump a ten rail fence.]

A very beautiful statuette came from Philadelphia. It was well modeled and represents genius and artistic handicraft in the sculptor. It exhibits Adam entirely nude—an anatomically correct man—upon the verge of a pond of water, where he has had the first glimpse of himself in nature's mirror. From the pond luxurious plants are springing, in the midst of which the figure is standing. It reminds one too much of Rogers' boy after the woodchuck, and one looks involuntarily for the dog or a glimpse of the game.

A beautiful water color comes from New York. The figure, pedestal, and bas-relief upon the front panel of the pedestal, are well drawn and show artistic talent of no mean order. Adam is simply represented in a well poised figure wearing an apron of fig-leaves. To the bas-relief is entrusted the duty of telling the story. This represents both Adam and Eve leaning against an apple tree, with a serpent shaking the tree. [We judge him to be shaking it, for the apples have fallen at their feet and there seems to be no other legitimate employment for the reptile.]

The other designs possess no particular merit, so we will not occupy space in attempting a description of them. When a design has been decided upon, we will acquaint our readers with it. One reason why a larger number of designs has not been received is, that it seems to be difficult to convince people at a distance that Elmira is really in earnest in erecting a monument to Adam. They seem to regard it as a huge joke, and are afraid of falling victims to it. We were never more in sober earnest in our lives, while the character of the men who are managing the enterprise should be a sufficient guarantee of its genuineness.

